# Early C-reactive protein kinetics predict outcomes in unresectable hepatocellular carcinoma treated with immune checkpoint inhibitors: a multicenter, observational study comparing different C-reactive protein kinetic definitions

**DOI:** 10.3389/fonc.2026.1889205

**Published:** 2026-08-20

**Authors:** Joshua Leutiger, Melanie Bathon, Christian Möhring, Max Ole Hubert, Catherine Leyh, Alexandra Emilia Schlaak, Katharina Mitzlaff, Laurenz Krämer, Christoph Neumann-Haefelin, Carolin Zimpel, Bertram Bengsch, Christoph Roderburg, Najib Ben Khaled, Cennet Sahin, Marie-Luise Berres, Maria A. Gonzalez-Carmona, Anna Lena Saborowski, Arndt Vogel, Arndt Weinmann, Dirk Waldschmidt, Gabriel Allo

**Affiliations:** 1 Department of Gastroenterology and Hepatology, University Hospital Cologne, Faculty of Medicine, University of Cologne, Cologne, Germany; 2 Department of Gastroenterology, Hepatology and Endocrinology, Hannover Medical School, Hannover, Germany; 3 Department I of Internal Medicine, University Hospital of Bonn, Bonn, Germany; 4 Department of Medicine II, University Hospital, Ludwig Maximilian University Munich, Munich, Germany; 5 Clinic for Gastroenterology, Hepatology and Infectious Diseases, University Hospital Düsseldorf, Medical Faculty of Heinrich Heine University Düsseldorf, Düsseldorf, Germany; 6 Department of Medicine II, Medical Center University of Freiburg, Freiburg, Germany; 7 Department of Internal Medicine 1, University Hospital Schleswig-Holstein, Lübeck, Germany; 8 Medical Department III, University Hospital Rheinisch-Westfälische Technische Hochschule Aachen (RWTH) Aachen, Aachen, Germany; 9 Department of Internal Medicine I, University Medical Center of the Johannes Gutenberg University Mainz, Mainz, Germany

**Keywords:** biomarkers, C-reactive protein, hepatocellular carcinoma, immune checkpoint inhibitors, immuno-oncology, liver cancer

## Abstract

**Background and aims:**

Immune checkpoint inhibitors (ICIs) have become the standard therapy for unresectable hepatocellular carcinoma (HCC). However, reliable prognostic biomarkers for treatment response remain warranted. This study assessed the prognostic value of early C-reactive protein (CRP) kinetics as an on-treatment biomarker in patients with hepatocellular carcinoma receiving ICIs.

**Methods:**

In this multicenter retrospective study, 289 patients with HCC treated with ICIs between 2019 and 2023 were analyzed. Patients were stratified using two established CRP kinetic models. The Fukuda model classified patients as flare responders, responders, or non-responders based on early CRP kinetics after treatment initiation. The Ishihara model categorized patients as “normal”, “normalized”, or “non-normalized” according to baseline and on-treatment CRP levels. Both models were additionally evaluated at eight weeks for earlier risk stratification.

**Results:**

According to the Fukuda model, flare responders demonstrated significantly prolonged median overall survival (34.5 vs 12.7 months), progression-free survival (12.42 vs 5.32 months), and higher objective response rates (53.7% vs 25.2%) compared with non-responders. In the Ishihara model, the normalized and normal groups showed significantly improved outcomes with median overall survival of 28.35 and 25.1 months, progression-free survival of 12.48 and 9.89 months, and objective response rates of 54.2% and 43.4%, respectively, compared with the non-normalized group (7.46 months; 3.35 months; 18.8%). The adapted 8-week Ishihara model enabled earlier risk stratification prior to first radiologic staging.

**Conclusions:**

Early C-reactive protein kinetics provide robust prognostic information in patients with HCC treated with ICIs. Both CRP-based models identify subgroups with significantly improved outcomes, with the Ishihara model enabling earlier identification of patients at high risk of treatment failure and thereby supporting earlier risk stratification during immunotherapy.

## Introduction

Primary liver cancer remains a major global health burden, ranking as the third leading cause of cancer-related mortality and the sixth most commonly diagnosed cancer worldwide as of 2022 (1). Hepatocellular carcinoma (HCC) accounts for the majority of primary liver cancers, comprising approximately 75 – 85% of cases, followed by intrahepatic cholangiocarcinoma (1).

The global incidence of HCC is projected to continue to rise, with annual diagnoses expected to exceed 1.4 million cases by 2040 and associated deaths reaching 1.3 million per year (2).

Many patients are diagnosed with HCC at an advanced, unresectable stage, leaving palliative systemic therapy as the only therapeutic option (3–6). For years, tyrosine kinase inhibitors (TKIs) such as sorafenib and lenvatinib served as the standard first-line treatment (7–9). Since 2020 novel therapeutic regimens combining immune checkpoint inhibitors (ICIs) with targeted agents, such as atezolizumab, a programmed death-ligand 1 (PD-L1) inhibitor, with bevacizumab, a vascular endothelial growth factor (VEGF) inhibitor, as well as dual ICI combinations including tremelimumab, an anti-cytotoxic T-lymphocyte-associated antigen 4 (CTLA-4) inhibitor with durvalumab (PD-L1 inhibitor), or ipilimumab (CTLA-4 inhibitor) with nivolumab (PD-1 inhibitor), have emerged as superior treatment options compared to TKIs (10–14). Despite superior survival, objective response rates (ORR) remain limited, ranging from 20% to 36%, and immune-related adverse events (irAEs) represent a clinical concern (10–17).

Several biomarkers predicting response to ICI therapy in solid tumors are established, including PD-L1 expression, tumor mutational burden (TMB), and microsatellite instability (MSI) (18–20), however, most of these biomarkers failed to reliably predict outcome in the overall population of patients with HCC (13, 19–22).

Systemic inflammation has been associated with poor outcomes in patients with HCC. In this context, inflammatory biomarkers, particularly C-reactive protein (CRP), have emerged not only as prognostic markers but also as potential predictors of ICI treatment response, thereby leading to the development of novel CRP-based risk models (23–25). While these models have demonstrated promising prognostic value in patients with HCC receiving ICIs, prospective validation is warranted (26–28).

Furthermore, dynamic changes in CRP levels following ICI initiation have emerged as a potential on-treatment biomarker for treatment response across several solid tumors (29–33). Fukuda et al. categorized patients with metastatic renal cell carcinoma (RCC) into three distinct CRP kinetic groups following ICI initiation and identified the subgroup of flare responders – characterized by an early doubling of CRP value within the first four weeks of treatment followed by a decline below baseline – as being most strongly associated with favorable tumor response and improved survival outcomes (29). Ishihara et al. proposed an alternative classification incorporating both baseline and on-treatment CRP measurements. In patients with metastatic RCC treated with nivolumab, low baseline CRP was associated with prolonged OS but not PFS. Notably, when patients with normal baseline CRP were combined with those exhibiting elevated baseline CRP who achieved CRP normalization within the first three months of therapy, these two groups were associated with significantly improved PFS, OS and ORR compared to patients with elevated baseline CRP who failed to achieve CRP normalization (34).

Based on these findings, the present study aimed to evaluate the prognostic value of these two models of early CRP dynamics in a large real-world cohort of patients with unresectable HCC treated with ICIs.

## Methods

### Study design and patient cohort

This national, retrospective, multicenter observational study included 289 patients aged 18 years or older from nine German tertiary centers, who began systemic therapy with ICIs for advanced HCC between January 1, 2019 and December 31, 2023.

**Figure 1:**
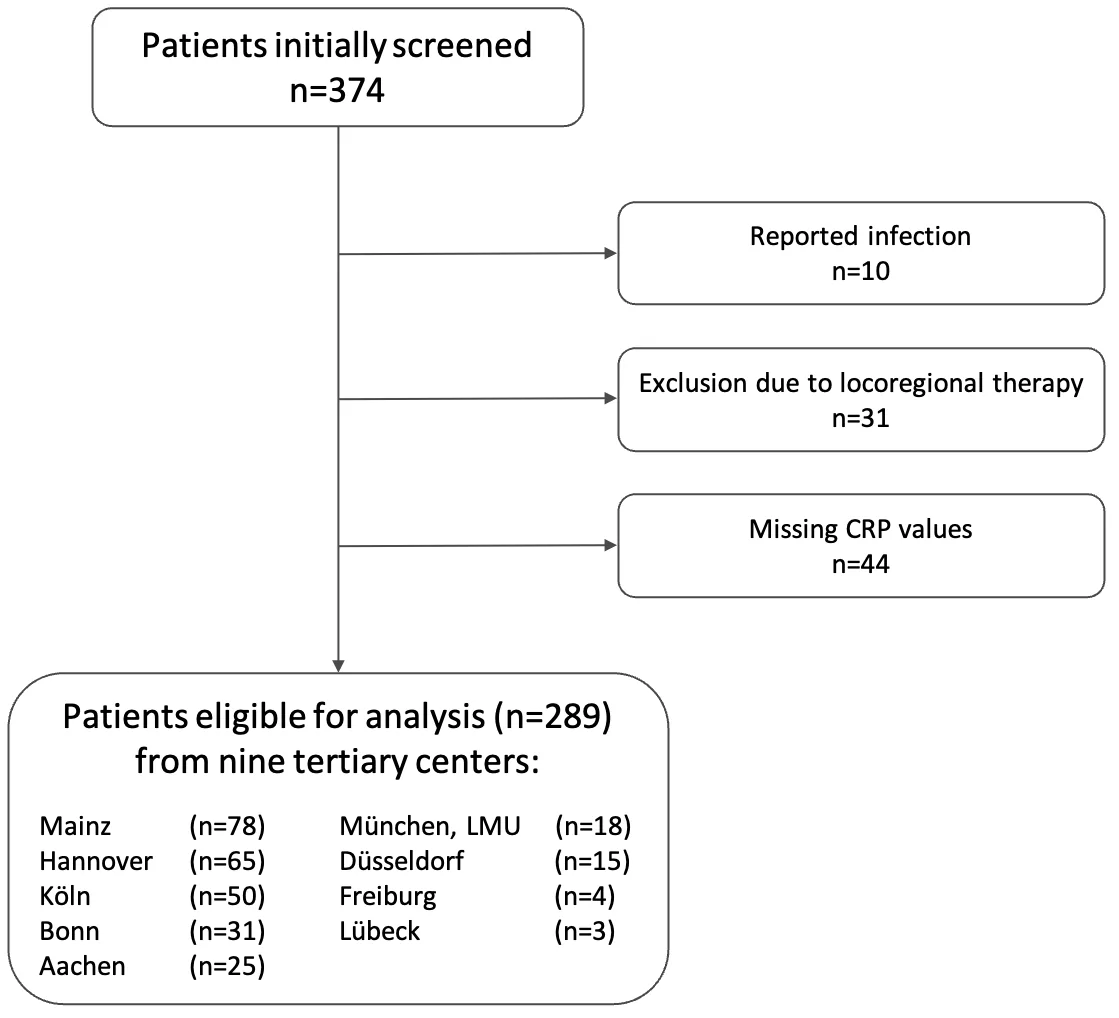
Flow chart of selection process.

Patients were excluded if they had previously undergone liver transplantation or received local ablative, surgical or radiotherapeutic procedures within ten days prior to or up to four weeks after initiation of ICI therapy, or had evidence of an acute infection within the first four weeks after therapy initiation, as determined by documented diagnosis of infection and/or initiation of antibiotic therapy in the electronic medical records (shown in **Figure 1**).

Baseline clinical characteristics and laboratory parameters were obtained from the electronic medical records. All participating centers used routine clinical CRP assays with an upper limit of normal (ULN) of 5 mg/L. CRP levels were measured at least once within 30 days prior to treatment initiation, during the first four weeks after treatment initiation and again within the three months following treatment initiation. As there are no standardized high-risk cut-off values for neutrophile-lymphocyte ratio (NLR) or alpha-fetoprotein (AFP), thresholds of NLR ≥2.7 and AFP ≥400 ng/mL – previously associated with poorer OS – were applied in the present study (35–37).

This study was performed in accordance with the Declaration of Helsinki. The study protocol was approved by the Ethics Committee of the Medical Faculty of the University of Cologne (reference: 23-1468-retro) and the respective institutional ethics committees. The need for written informed consent was waived by the Ethics Committee of the Medical Faculty of the University of Cologne due to the retrospective design and the use of anonymized data.

### Definition of CRP kinetic groups

In alignment with the definitions proposed by Fukuda et al. and Ishihara et al., patients were stratified into three different groups based on CRP dynamics within the first three months after ICI initiation (29, 34).

Following the definition by Fukuda et al., flare responders were characterized by doubling of baseline CRP levels within the first four weeks with a subsequent decline below baseline within the first three months after treatment initiation. The subgroup of responders demonstrated a CRP decline ≥30% from baseline at least once within three months, without meeting the flare definition. All remaining patients were classified as non-responders.

In reference to the model proposed by Ishihara et al., patients with baseline CRP levels ≤5 mg/L were categorized as “normal”. Those with initially elevated CRP levels and a subsequent decline below 5 mg/L within the first three months were classified as “normalized”. Patients with persistently elevated CRP levels throughout this period were classified as “non-normalized”. A CRP threshold of ≤5 mg/L was selected instead of the 10 mg/L cut-off applied in the original publication by Ishihara et al. (34), as 5 mg/L corresponds to the ULN according to the laboratory reference ranges of the participating centers.

### Study endpoints

The primary endpoint was OS, with PFS and ORR assessed as secondary endpoints. OS was defined as the time from therapy initiation to death from any cause. Patients lost to follow-up were censored at the date of last clinical contact. PFS was defined as the time from initiation of ICI therapy to clinical or radiologically confirmed disease progression or death from any cause. Radiological assessments were performed prior to treatment initiation and subsequently every 8–12 weeks using computed tomography (CT) or magnetic resonance imaging (MRI). Tumor response to ICI therapy was evaluated investigator-based in analogy to the Response Evaluation Criteria in Solid Tumors (RECIST), version 1.1, with outcomes classified as complete response (CR), partial response (PR), stable disease (SD) and progressive disease (PD) (38). ORR was defined as the proportion of patients achieving a best overall response of CR or PR during ICI therapy. Death prior to first radiological assessment was classified as PD.

### Statistical analysis

All statistical analyses were performed using R Statistical Software version 4.4.2 (R Foundation for Statistical Computing, Vienna, Austria) with the packages *survival*, *survminer* and *finalfit*. OS and PFS were estimated with the univariable Kaplan-Meier analysis and tested with the log-rank test. In addition, pairwise log-rank tests were performed between the CRP kinetic groups, with adjustment for multiple comparisons using the Holm method. Median follow-up was calculated using the reverse Kaplan-Meier method. Differences in baseline characteristics across CRP kinetic groups were assessed using the Kruskal-Wallis test for continuous variables and the Pearson chi-square test or Fisher’s exact test for categorical variables, as appropriate. Pairwise comparisons of best overall response distributions across CRP kinetic groups were performed using the Pearson chi-square test or Fisher’s exact test, as appropriate, with adjustment for multiple comparisons using the Holm method. Logistic regression analysis was used to assess predictors of ORR, incorporating CRP groups and clinically relevant covariates. Univariate and multivariate Cox regression models were applied to evaluate the prognostic impact of early CRP kinetics alongside baseline characteristics on OS and PFS. Variables demonstrating a statistically significant association in univariate analysis were included in the multivariate Cox regression. Baseline CRP was not included in multivariate analyses due to its close correlation with the CRP group definitions proposed by Ishihara et al.

Categorical variables were presented as frequency and percentage, continuous variables as median, range and interquartile ranges (IQR), as appropriate. All statistical tests were two-sided and p-values <0.05 were considered statistically significant.

## Results

### Baseline characteristics of patient cohort

The median age at initiation of ICI therapy among the 289 included patients was 68 years (range 28 – 91), with a predominance of male patients (83%). Underlying liver cirrhosis was present in 95.2% of cases, most commonly related to viral hepatitis (42.6%), followed by alcohol-associated liver disease (28.7%). Liver function was generally preserved, as reflected by the high proportion of patients with Child-Pugh Stadium A (72.6%). Eastern Cooperative Oncology Group (ECOG) performance status was 0–1 in 92.9% of the cohort. At baseline, extrahepatic spread was observed in 33.2% of patients. Prior to systemic therapy, 53.8% of patients (n=155) had received locoregional therapies, including transarterial chemoembolization (TACE), microwave ablation (MWA), radiofrequency ablation (RFA), selective internal radiation therapy (SIRT), radiotherapy, or surgical resection. The ICIs administered were atezolizumab with bevacizumab (81.3%), tremelimumab with durvalumab (8.7%), nivolumab (5.2%), pembrolizumab (4.2%) and other agents (0.7%). The median number of ICI cycles administered was 7 (IQR: 4 – 15). The first radiological assessment was conducted at a median of 80 days (IQR: 64 – 96) following initiation of ICI treatment. Median baseline serum AFP and CRP levels were 41 ng/mL (IQR: 7.1–681 ng/mL) and 7.3 mg/L (IQR: 3.2 – 22.7 mg/L), respectively.

With regard to the clinical outcomes, median OS was 11.96 months (IQR: 4.93 – 22.14), and median PFS was 5.62 months (IQR: 2.99 – 13.11). Using the reverse Kaplan-Meier method, median follow-up was 27.46 months (IQR: 15.14 – 41.16). The ORR was 33.8%, and the DCR was 65.5%. A detailed overview of baseline characteristics is provided in **Table 1**.

**Table 1:** Baseline characteristics of the cohort.

Baseline characteristic	Level	Value
Age – yr	Median (range)	68 (28–91)
Gender – no. (%)	Female	49 (17%)
	Male	240 (83%)
ECOG PS – no. (%)	0	115 (50.9%)
	1	95 (42%)
	2	14 (6.2%)
	3	2 (0.9%)
Cirrhosis – no. (%)	Yes	238 (95.2%)
	No	12 (4.8%)
Etiology – no. (%)	Viral hepatitis	107 (42.6%)
	Alcohol	72 (28.7%)
	MASLD	44 (17.5%)
	Cryptogenic	19 (7.6%)
	Other	4 (1.6%)
	PBC	3 (1.2%)
	Hemochromatosis	1 (0.4%)
	AIH	1 (0.4%)
Child Pugh Score – no. (%)	A/5	138 (51.1%)
	A/6	58 (21.5%)
	B/7	48 (17.8%)
	Other	26 (9.6%)
Extrahepatic Manifestation – no. (%)	Yes	96 (33.2%)
	No	193 (66.8%)
Prior Local Treatment* – no. (%)	Yes	155 (53.8%)
	No	133 (46.2%)
Medication – no. (%)	Atezolizumab/Bevacizumab	235 (81.3%)
	Tremelimumab/Durvalumab	25 (8.7%)
	Nivolumab	15 (5.2%)
	Pembrolizumab	12 (4.2%)
	Other	2 (0.7%)
Baseline AFP – ng/mL	Median (IQR)	41 (7.1–681)
Baseline CRP – mg/L	Median (IQR)	7.3 (3.2–22.7)
Baseline NLR	Median (IQR)	3.4 (2.1–5.3)

*Local treatment modalities included transarterial chemoembolization (TACE), microwave ablation (MWA), radiofrequency ablation (RFA), selective internal radiation therapy (SIRT), radiotherapy, and surgical resection.

AIH, autoimmune hepatitis; AFP, alpha-fetoprotein; CRP, C-reactive protein; ECOG PS, Eastern Cooperative Oncology Group performance status; MASLD, metabolic dysfunction associated steatotic liver disease; NLR, neutrophile-lymphocyte ratio; PBC, primary biliary cholangitis.

### Survival outcomes and tumor response according to the Fukuda model

Based on the classification by Fukuda et al., 41 (14.2%) patients were classified as flare responders, 95 (32.9%) as responders and 153 (52.9%) as non-responders. Baseline characteristics were balanced across these groups, except for significantly higher baseline CRP levels in the responder group compared to flare responders and non-responders (**Supplementary Table 1**).

**Figure 2:**
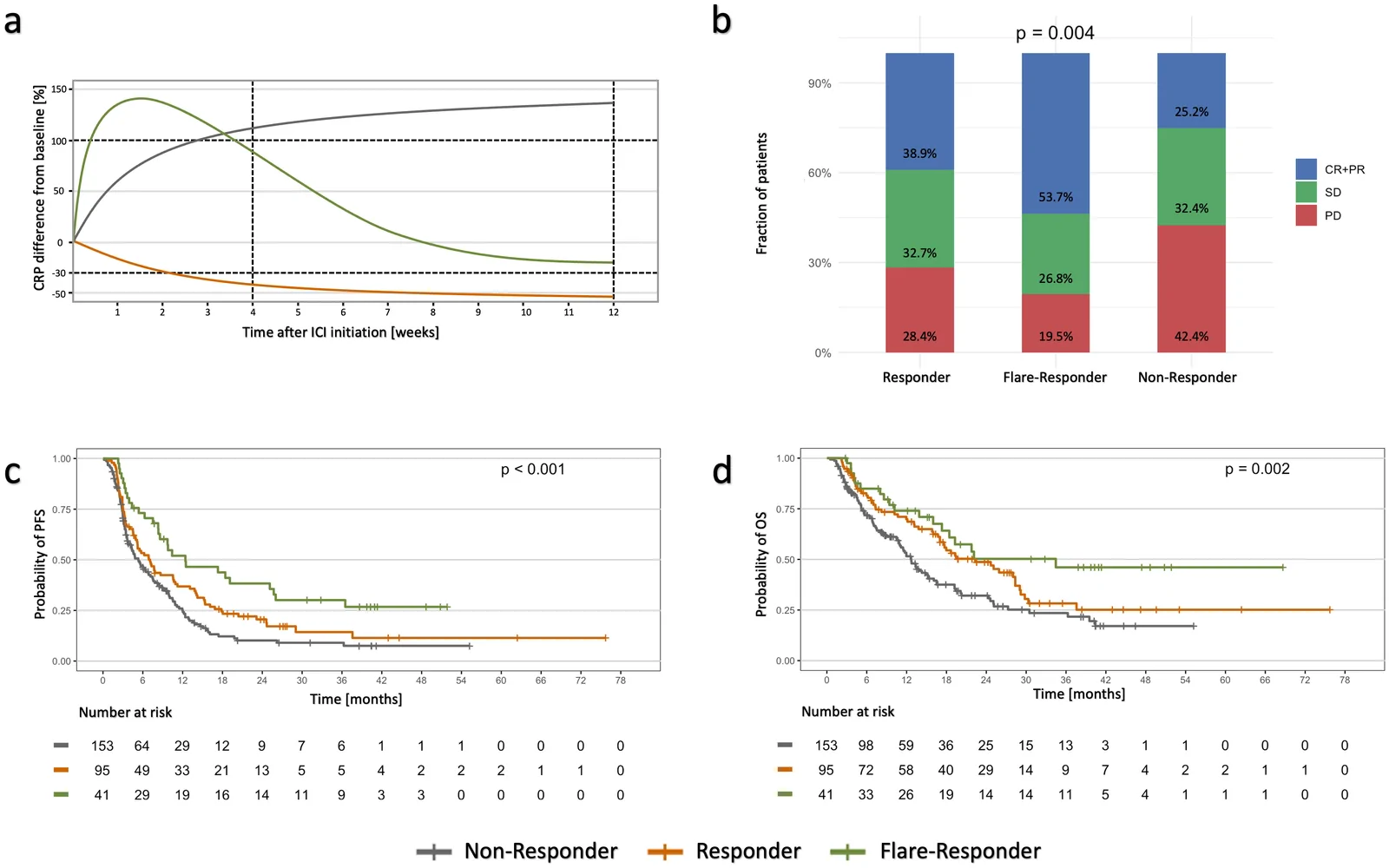
Clinical outcomes according to the Fukuda model. **(a)** Schematic illustration of the CRP groups defined by Fukuda et al. **(b)** Tumor response at first radiological assessment. In the responder group, there were 8 complete response (CR), 29 partial response (PR), 31 stable disease (SD) and 27 progressive disease (PD). In the flare responder group, there were 4 CR, 18 PR, 11 SD and 8 PD. In the non-responder group, there were 3 CR, 35 PR, 49 SD and 64 PD. Kaplan-Meier plots for **(c)** progression-free survival (PFS) and **(d)** overall survival (OS). P-values were calculated using the log-rank test for survival analyses and the chi-square test for response distributions. Abbreviations: CRP, C-reactive protein; ICI, Immune-checkpoint inhibitors.

PFS differed significantly among the three groups (p<0.001). Flare responders demonstrated the longest median PFS at 12.42 months (95%-CI 8.34 – 26.02 months), followed by responders at 7.1 months (95%-CI 5.12 – 10.91 months) and non-responders at 5.32 months (95%-CI 4.04 – 7.46 months). Similarly, median OS was significantly prolonged (p=0.002) with 34.5 months (95%-CI 18.4 months – “not reached”) in the flare responder group, compared to 22.3 months (95%-CI 17–29 months) in responders and 12.7 months (95%-CI 10.9 – 16.1 months) in non-responders (shown in **Figure 2**). Pairwise comparisons demonstrated significant differences between all three groups for PFS (flare-responder vs. non-responder: adjusted p<0.001; responder vs. non-responder: adjusted p=0.043; flare-responder vs. responder: adjusted p=0.043). For OS, both flare responders and responders showed significantly longer OS compared with non-responders (adjusted p=0.008 and p=0.031, respectively), whereas no significant difference was observed between flare responders and responders (adjusted p=0.133). In univariate Cox regression analysis, both flare responders and responders were significantly associated with longer PFS and OS compared to non-responders. After multivariate adjustment, however, only the flare responder group remained independently associated with prolonged PFS (HR 0.44, 95% CI [0.26 – 0.75], p=0.003) and OS (HR: 0.41, 95% CI [0.21 – 0.82], p=0.011) (shown in **Table 2**; detailed univariate and multivariate analyses are provided in **Supplementary Tables 3**, **4**).

**Table 2:** Univariate and multivariate Cox Regression analyses for OS and PFS.

CRP kinetic model	CRP response group	n	PFS				OS			
			Univariate		Multivariate		Univariate		Multivariate	
			HR (95% CI)	p	HR (95% CI)	p	HR (95% CI)	p	HR (95% CI)	p
Fukuda Model	Non-Responder	153								
	Responder	95	0.74 [0.56 – 0.99]	0.044*	0.84 [0.56 – 1.25]	0.393	0.66 [0.47 – 0.93]	0.016*	0.75 [0.46 – 1.22]	0.253
	Flare Responder	41	0.46 [0.3 – 0.7]	<0.001***	0.44 [0.26 – 0.75]	0.003**	0.45 [0.27 – 0.74]	0.002**	0.41 [0.21 – 0.82]	0.011*
Ishihara Model	Non-Normalized	133								
	Normal	108	0.51 [0.38 – 0.68]	<0.001***	0.45 [0.31 – 0.65]	<0.001***	0.33 [0.23 – 0.47]	<0.001***	0.32 [0.2 – 0.52]	<0.001***
	Normalized	48	0.41 [0.28 – 0.6]	<0.001***	0.37 [0.22 – 0.6]	<0.001***	0.29 [0.18 – 0.46]	<0.001***	0.29 [0.16 – 0.52]	<0.001***

Adjusted for: Child Pugh Score, ECOG PS, ICI regimen, baseline AFP and baseline NLR.

Univariate and multivariate Cox proportional hazards models were used to evaluate factors associated with OS and PFS. Variables demonstrating a statistically significant association in univariate analyses or were considered clinically relevant were included in the multivariate models (baseline CRP was not included due to its close correlation with the CRP group definitions proposed by Ishihara et al.). For clarity, only CRP response groups are listed in this table; detailed results are provided in **Supplementary Tables 3** and **4**.

AFP, alpha-fetoprotein; CRP, C-reactive protein; ECOG PS, Eastern Cooperative Oncology Group performance status; HR, hazard ratio; NLR, neutrophile-lymphocyte ratio; OS, overall survival; PFS, progression-free survival.

With regard to tumor response, ORR differed significantly between the groups (p=0.004). Flare responders exhibited the highest ORR at 53.7%, whereas responders achieved an ORR of 38.9% and non-responders of 25.2% (shown in **Figure 2**). Pairwise comparisons showed a significantly higher ORR only for flare responders compared with non-responders (adjusted p=0.004), whereas the remaining pairwise comparisons were not statistically significant. In univariate logistic regression analysis, both responders and flare responders were significantly associated with ORR. However, after multivariate adjustment, only flare responders remained independently associated with ORR (OR 3.3, 95% CI 1.36 – 8.27, p=0.009) (**Supplementary Table 5**).

### Survival outcomes and tumor response according to the Ishihara model

Using the classification by Ishihara et al., 108 (37.4%) patients were classified as “normal”, 48 (16.6%) patients as “normalized”, and 133 (46%) as “non-normalized”. Significant differences were observed in several baseline characteristics. Impaired liver function (Child Pugh >A) and higher baseline AFP levels were more frequent among non-normalized patients, compared to the normal group. Baseline CRP levels were lowest in the normal group, consistent with the CRP-based definition of groups. Furthermore, the distribution of ICI regimens varied significantly between the groups (**Supplementary Table 2**).

**Figure 3:**
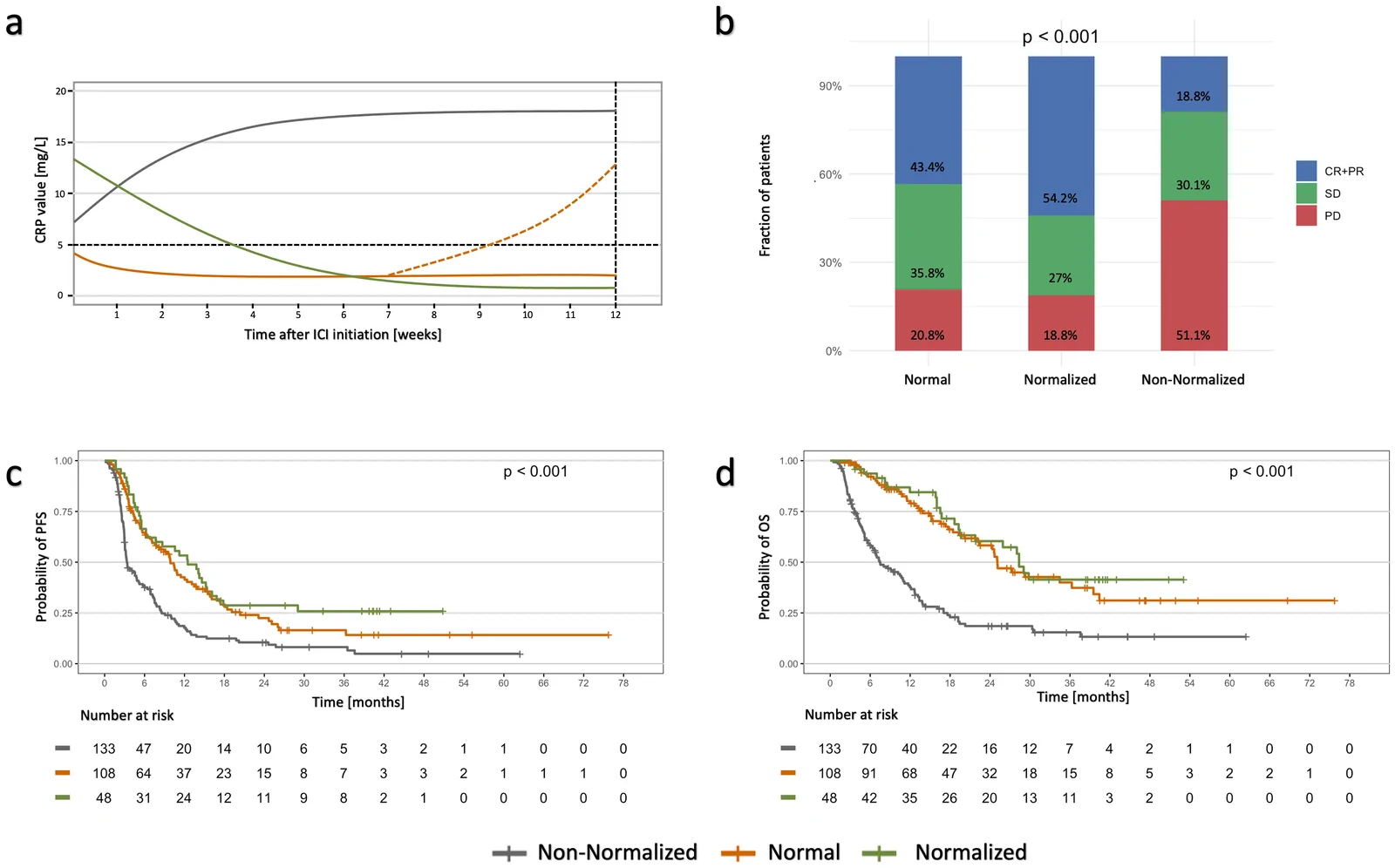
Clinical outcomes according to the Ishihara model. **(a)** Schematic illustration of the CRP groups defined by Ishihara et al. **(b)** Tumor response at first radiological assessment. In the normal group, there were 5 complete response (CR), 41 partial response (PR), 38 stable disease (SD) and 22 progressive disease (PD). In the normalized group, there were 6 CR, 20 PR, 13 SD and 9 PD. In the non-normalized group, there were 4 CR, 21 PR, 40 SD and 68 PD. Kaplan-Meier plots for **(c)** progression-free survival (PFS) and **(d)** overall survival (OS). P-values were calculated using the log-rank test for survival analyses and the chi-square test for response distributions. Abbreviations: CRP, C-reactive protein; ICI, Immune checkpoint inhibitors.

PFS differed significantly across groups (p<0.001). Median PFS was longest in the normalized group at 12.48 months (95%-CI 6.27 – 16.85 months), followed by the normal group at 9.89 months (95%-CI 7.59 – 13.5 months) and the non-normalized group at 3.35 months (95%-CI 3.06 – 5.12 months). Similarly, OS was significantly prolonged in the normalized and normal groups (p<0.001) with a median OS of 28.35 months (95%-CI 19.45 months – “not reached”) for the normalized and 25.1 months (95%-CI 22.34 – 40.4 months) for the normal group compared to 7.46 months (95%-CI 6.14 – 11.1 months) in the non-normalized group (shown in **Figure 3**). Pairwise comparisons demonstrated significantly longer PFS and OS for both the normalized and normal groups compared with the non-normalized group (all adjusted p<0.001), whereas no significant difference was observed between the normalized and normal groups (PFS: adjusted p=0.251; OS: adjusted p=0.444). In univariate Cox regression, both the normalized and normal groups were significantly associated with improved PFS and OS compared to the non-normalized group. These associations remained significant after multivariate adjustment (shown in **Table 2**, detailed univariate and multivariate analyses are provided in **Supplementary Tables 3**, **4**). In additional multivariate analyses replacing the Ishihara CRP kinetics classification with dichotomized baseline CRP (≤5 vs. >5 mg/L), baseline CRP remained independently associated with OS, PFS, and ORR (**Supplementary Table 6**).

ORR also differed significantly between the groups (p<0.001) and reflected the patterns of survival outcomes. The normalized group achieved the highest ORR (54.2%), followed by the normal group (43.4%) and the non-normalized group (18.8%) (shown in **Figure 3**). Pairwise comparisons showed significantly higher ORR in both the normalized and normal groups compared with the non-normalized group (both adjusted p<0.001), whereas no significant difference was observed between the normalized and normal groups (adjusted p=0.438). Both normalized and normal groups were significantly associated with higher ORR in univariate logistic regression and remained independent predictors after multivariate adjustment (OR 5.89 [2.33 – 15.66], p<0.001; OR 4.63 [2.1 – 10.87], p<0.001) (**Supplementary Table 5**).

### Earlier prognostic stratification using 8-week adjustment

To assess whether earlier CRP kinetics could refine risk stratification, both models were reevaluated using CRP measurements obtained within the first eight weeks after initiation of therapy.

**Figure 4:**
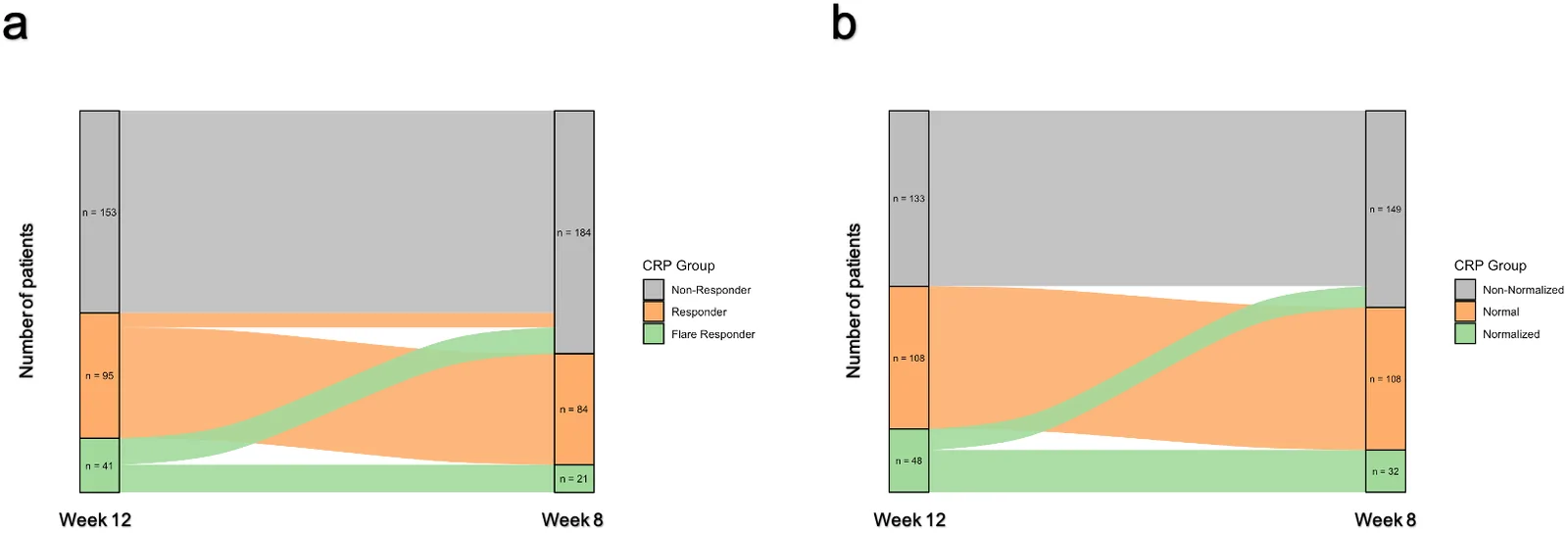
Distribution of patients across CRP response groups at 8-week and 12-week assessments. Sankey plots illustrating the reassignment of patients to CRP response groups eight weeks after initiation of therapy. **(a)** Fukuda model: 21 of 41 flare responders (51.2%) were identified at week eight. **(b)** Ishihara model: 32 of 48 patients (66.7%) in the normalized group were already classified at week eight. Abbreviations: CRP, C-reactive protein.

**Figure 5:**
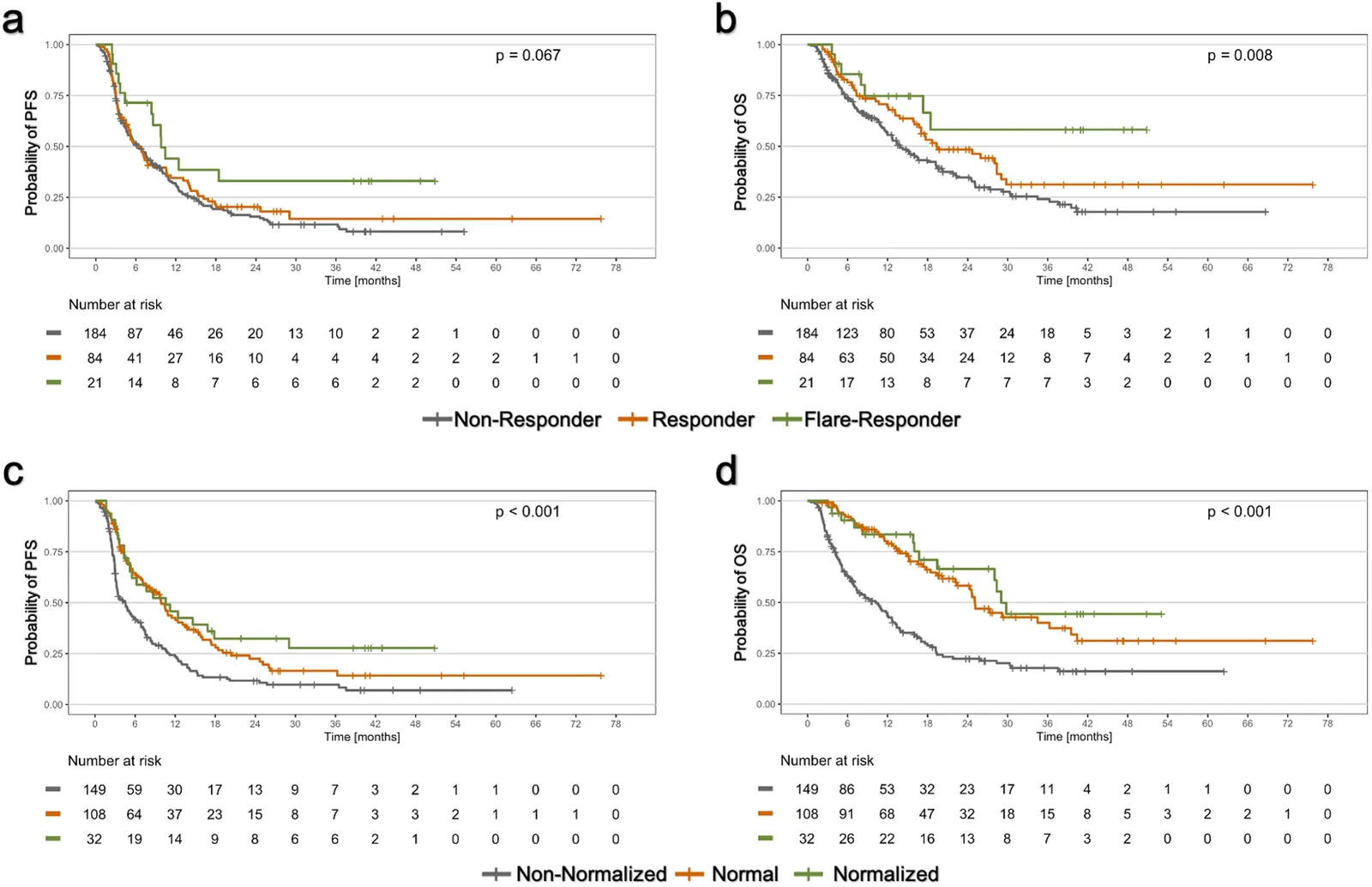
Survival outcomes at eight weeks after ICI initiation. Kaplan-Meier plots for **(a)** progression-free survival (PFS) and **(b)** overall survival (OS) stratified according to the Fukuda model at eight weeks after initiation of immune checkpoint inhibitor (ICI) therapy. Kaplan-Meier plots for **(c)** PFS and **(d)** OS stratified according to the Ishihara model at eight weeks after ICI initiation. P-values were calculated using the log-rank test.

When applying the Fukuda model using the 8-week assessment, only 51.2% of the original flare responders could be identified at this earlier time point (shown in **Figure 4**), and significant differences among groups were observed only with respect to OS (p=0.008). Median OS was “not reached” (95%-CI 17.3 months – “not reached”) in flare responders, 19.4 months (95%-CI 16.7–29 months) in responders and 13.9 months (95%-CI 11.7 – 19.2 months) in non-responders. Median PFS no longer differed significantly between groups (p=0.067), reaching 9.79 months (95%-CI 8.34 months – “not reached”) in flare responders, 6.21 months (95%-CI 4.86 – 10.58 months) in responders and 6.37 months (95%-CI 4.76 – 8.48 months) in non-responders (shown in **Figure 5**). Similarly, ORR did not differ significantly across groups (p = 0.09), with an ORR of 57.1%, 38.1%, and 29.1% in flare responders, responders and non-responders, respectively.

In contrast, when applying the Ishihara model, the prognostic performance of the 8-week assessment was comparable to that of the 3-month evaluation at the time of first radiological staging. Within eight weeks, 66.7% of patients in the normalized group achieved a decline in CRP levels below the ULN (shown in **Figure 4**). PFS remained significantly different among the groups (p<0.001), with a median PFS of 10.58 months (95%-CI 5.52 months – “not reached”) in the normalized group, 9.89 months (95%-CI 7.59 –13.5 months) in the normal group, and 4.53 months (95%-CI 3.25 – 6.27 months) in the non-normalized group. OS showed similar significant differences (p<0.001), with median OS of 29 months (95%-CI 28.02 months – “not reached”), 25.1 months (95%-CI 22.34 – 40.4 months), and 10.2 months (95%-CI 7.29 – 12.7 months) for “normalized”, “normal” and “non-normalized”, respectively (shown in **Figure 5**). ORR also differed significantly between groups (p<0.001), reaching 53.1% in the normalized group, 43.4% in the normal group, and 22.8% in the non-normalized group.

## Discussion

This multicenter study demonstrates the prognostic relevance of early CRP kinetics following initiation of ICI therapy in patients with unresectable HCC. Both CRP-based models proposed by Fukuda et al. and Ishihara et al. consistently met the clinical endpoints, showing significant association with improved OS, PFS and ORR. In multivariate analyses, particularly flare responders according to the Fukuda model as well as patients classified as “normalized” or “normal” according to the Ishihara model emerged as independent predictors of survival outcomes and tumor response. The Ishihara model, in particular, enables early identification of patients with unfavorable CRP kinetics as early as eight weeks after treatment initiation. These findings support the concept that on-treatment assessment of CRP kinetics may serve as a widely available and cost-effective biomarker to support individualized therapeutic decision-making and timely treatment adaption.

Our findings regarding the flare phenomenon are consistent with previous reports across various tumor entities. First described by Fukuda et al., the CRP flare response has demonstrated prognostic value in several solid tumors, including RCC, urothelial carcinoma (UC) and non-small cell lung cancer (NSCLC) (29–33). The Ishihara model, originally developed in patients with metastatic RCC treated with ICIs, has been less extensively studied (34, 39). To our knowledge, this study represents the first evaluation of the Ishihara model in HCC. Thus, our findings extend previous observations to a large, real-world European cohort of patients with unresectable HCC treated with ICI-based combination regimens.

Comparative analysis of both CRP kinetic models suggests differences in their clinical applicability. Within the Ishihara model, lack of CRP normalization (“non-normalized”) may indicate a higher risk of unfavorable outcomes and thus support earlier staging and timely treatment modification. This interpretation was further supported by the pairwise analyses, which consistently demonstrated significant differences between the non-normalized group and both the normalized and normal groups with regard to OS, PFS, and ORR, whereas no significant differences were observed between the normalized and normal groups for any of the study endpoints. In contrast, the Fukuda model identified a distinct subgroup of flare responders with particularly favorable outcomes compared to non-responders. However, when responders were compared to non-responders, the model failed to demonstrate independent prognostic significance in multivariate analyses. Furthermore, pairwise analyses showed a less consistent pattern across endpoints, with a pattern comparable to that of the Ishihara model observed only for OS. Pairwise comparisons for PFS and ORR, however, differed, indicating a less stable risk stratification across these clinically relevant outcomes. Consequently, the model’s overall prognostic ability for a favorable disease course appears to be largely driven by the subgroup of patients identified as flare responders, who accounted for only 14.2% of our cohort.

To date, only one single-center retrospective study from Asia has suggested that CRP flare response may represent a prognostic marker in patients with unresectable HCC treated with ICIs (33). However, this study included patients receiving ICIs in combination with locoregional therapy. Our present multicenter European study analyzed patients with unresectable HCC who were predominantly treated with current first-line ICI-based regimens (95.2%). Another important methodological strength of our study was the exclusion of patients who received locoregional therapies shortly before or within 30 days after the initiation of ICI treatment, as previous studies have shown that CRP levels typically rise in the first week after locoregional therapy and normalize within 30 days thereafter (40, 41). This approach reduces confounding due to procedure-related inflammation and distinguishes our work from the previous single-center analysis (33). Nevertheless, the Fukuda model has demonstrated prognostic value in patients with HCC treated with a combination of TACE, lenvatinib and ICIs (42), suggesting that on-treatment CRP kinetics may have broader applicability beyond systemic therapy with ICIs.

With regard to individual ICI regimens, adjustment for treatment regimen in the multivariate analyses identified ICI regimen as an independent factor associated with OS. However, this finding should be interpreted primarily in the context of adjustment for a potential confounder rather than as evidence supporting regimen-specific treatment effects, as our study was not designed or powered to compare the efficacy of individual ICI regimens, and the distribution of treatment regimens was markedly unbalanced.

The 8-week adaptation of the Ishihara model highlights its potential clinical utility, as significant associations with PFS and OS were already observed prior to first radiological staging. Early identification of patients with unfavorable CRP kinetics could support earlier radiological reassessment and treatment modification (43, 44).

Notably, in RCC, UC and NSCLC dynamic, on-treatment changes in CRP-based risk scores have been shown to identify patients who derive survival benefit from treatment beyond radiologic progression (TBP) (45). Although our study did not specifically assess CRP kinetics in the context of TBP, these findings underscore the potential value of dynamic CRP assessment in guiding individualized treatment strategies. Nevertheless, prospective validation is essential before CRP-guided treatment adaptations can be implemented into routine clinical practice.

Elevated baseline CRP values have been associated with poor prognosis in HCC. Conversely, previous studies have demonstrated significantly longer PFS and OS in patients with lower baseline CRP receiving ICI therapy (41, 46, 47). Consistent with these observations, our data confirm that lower baseline CRP levels were independently associated with improved OS, PFS, and higher ORR. However, longitudinal assessment of CRP kinetics provides additional prognostic information beyond the baseline measurement alone. Notably, patients with initially elevated CRP levels that normalized during the first three months of treatment (normalized group) achieved outcomes comparable to those of patients with baseline CRP levels ≤5 mg/L (normal group), suggesting that on-treatment CRP kinetics provide prognostic information beyond that obtained from a single baseline CRP measurement at therapy initiation.

The exact mechanisms linking CRP kinetics to ICI efficacy have not yet fully been understood. Chronic inflammation may induce an immunosuppressive tumor microenvironment (TME), thereby potentially facilitating tumor progression (48, 49). Elevated CRP levels have been associated with increased infiltration of regulatory T cells and M2 macrophages in RCC, supporting the concept that chronic inflammation indicated by CRP levels may reflect an immunosuppressive TME (50). Moreover, it has been hypothesized, that local inflammatory conditions within the TME could promote resistance to ICI therapy and enable immune escape (51, 52). However, an early elevation in CRP levels following ICI initiation – as observed in patients classified as flare responders – could indicate an early immune activation and dynamic remodeling of TME (29, 53). Further translational research is needed in order to examine how these immunologic processes relate to distinct CRP kinetic patterns in the context of ICI therapy.

Several limitations of this study should be acknowledged. Since CRP is a non-specific inflammatory marker, it remains crucial to distinguish between tumor-related and infection-related elevations in CRP. To minimize bias from infectious complications, patients with documented infections or antibiotic treatment within four weeks of ICI initiation were excluded. Nevertheless, subclinical infections cannot be entirely ruled out, and misclassification due to infection-related increase in CRP levels may have occurred. The observed association of CRP kinetics with tumor-related endpoints like ORR, however, suggests that the findings were not primarily driven by uncontrolled infections. In addition, the retrospective design of this study introduces several inherent limitations. First, CRP measurements were obtained at non-standardized intervals, which may have resulted in misclassification of patients into the different CRP kinetic patterns. Second, 44 patients were excluded because of missing CRP values, which may have introduced the possibility of selection bias. Although this exclusion cannot be avoided in retrospective analyses of biomarker kinetics, the multicenter design including nine tertiary centers and the large overall cohort reduce the likelihood that the findings are driven by center-specific patient selection. Finally, analyses based on longitudinal biomarker kinetics are susceptible to immortal time bias, as patients must survive long enough to fulfill the criteria for specific kinetic response patterns. However, the Ishihara model already demonstrated prognostic value as early as eight weeks after treatment initiation, and only 8 of the 289 patients (2.8%) had a follow-up shorter than eight weeks. Although immortal time bias cannot be completely excluded, these findings suggest that its potential impact on our results is likely limited.

Despite these limitations, the large sample size and multicenter design involving nine tertiary centers strengthen the robustness of our findings.

In conclusion, this multicenter study supports early CRP kinetics as a valuable prognostic biomarker in patients with unresectable HCC treated with ICI-based regimens. Among the evaluated classifications, the Ishihara model – including its 8-week adaptation – emerged as particularly robust and clinically applicable. Its accessibility, low cost, and potential to complement radiologic assessment, indicate that early CRP kinetics may contribute to more individualized treatment strategies. Prospective studies with standardized intervals for CRP measurement are warranted to validate these findings and to further determine the optimal integration of CRP kinetics into clinical decision-making.

## Data Availability

The datasets presented in this article are not readily available because of institutional and ethical restrictions related to the multicenter study design. However, the analyzed data may be made available from the corresponding author upon reasonable request and with permission of the participating centers. Requests to access the datasets should be directed to Joshua Leutiger, joshua.leutiger@uk-koeln.de.
