# Supplementary material for: Early C-reactive protein kinetics predict outcomes in unresectable hepatocellular carcinoma treated with immune checkpoint inhibitors: a multicenter, observational study comparing different C-reactive protein kinetic definitions
Source: Front Oncol. 2026 Aug 20;16:1889205. doi: 10.3389/fonc.2026.1889205 (PMC13537936; doi:10.3389/fonc.2026.1889205)
Supplement: Supplementary file 1 [file Table1.docx]

**Supplementary material:**

Supplementary Table 1: Baseline characteristics according to Fukuda model

|  |  | **n** | **Non-Responder** | **Responder** | **Flare Responder** | **p** |
| --- | --- | --- | --- | --- | --- | --- |
| Total Number |  |  | 153 | 95 | 41 | NA |
| Age | Median (Range) |  | 69 (28-87) | 70 (42-85) | 65 (34-91) | 0.331 |
| Gender | Female | 49 | 25 (16.3%) | 18 (18.9%) | 6 (14.6%) | 0.779 |
|  | Male | 240 | 128 (83.7%) | 77 (81.1%) | 35 (85.4%) |  |
| ECOG PS | 0 | 115 | 63 (52.9%) | 33 (47.1%) | 19 (51.4%) | 0.465 |
|  | 1 | 95 | 45 (37.8%) | 34 (48.6%) | 16 (43.2%) |  |
|  | 2 | 14 | 10 (8.4%) | 3 (4.3%) | 1 (2.7%) |  |
|  | 3 | 2 | 1 (0.8%) | NA | 1 (2.7%) |  |
| Child Pugh Score | A | 196 | 101 (69.7%) | 67 (76.1%) | 28 (75.7%) | 0.782 |
|  | ≥B | 74 | 44 (39.3%) | 21 (23.9%) | 9 (24.3%) |  |
| Extrahepatic Manifestation | Yes | 96 | 45 (29.4%) | 39 (41.1%) | 12 (29.3%) | 0.144 |
|  | No | 193 | 108 (70.6%) | 56 (58.9%) | 29 (70.7%) |  |
| Prior Local Treatment | Yes | 155 | 78 (51.3%) | 52 (54.7%) | 25 (61%) | 0.534 |
|  | No | 133 | 74 (48.7%) | 43 (45.3%) | 16 (39%) |  |
| ICI | Atezolizumab/ Bevacizumab | 235 | 129 (84.3%) | 73 (76.8%) | 33 (80.5%) | 0.507 |
|  | Tremelimumab/Durvalumab | 25 | 13 (8.5%) | 9 (9.5%) | 3 (7.3%) |  |
|  | Nivolumab | 15 | 6 (3.9%) | 7 (7.4%) | 2 (4.9%) |  |
|  | Pembrolizumab | 12 | 5 (3.3%) | 4 (4.2%) | 3 (7.3%) |  |
|  | Other | 2 | NA | 2 (2.1%) | NA |  |
| Baseline AFP | >400 ng/mL | 77 | 37 (26.1%) | 28 (30.8%) | 12 (33.3%) | 0.596 |
|  | ≤400 ng/mL | 192 | 105 (73.9%) | 63 (69.2%) | 24 (66.7%) |  |
| Baseline CRP | >5 mg/L | 181 | 84 (54.9%) | 72 (75.8%) | 25 (61%) | 0.004** |
|  | ≤5 mg/L | 108 | 69 (45.1%) | 23 (24.2%) | 16 (39%) |  |
| Baseline NLR | ≥2.7 | 170 | 93 (65.5%) | 54 (64.3%) | 23 (59%) | 0.772 |
|  | <2.7 | 95 | 49 (34.5%) | 30 (35.7%) | 16 (41%) |  |

Data are Median (Range) or n (%). P-values were calculated using Kruskal-Wallis test for continuous variables and the Pearson chi-square test or Fisher’s exact test for categorical variables.

Abbreviations: AFP, alpha-fetoprotein; CRP, C-reactive protein; ECOG PS, Eastern Cooperative Oncology Group performance status; ICI, immune checkpoint inhibitor; NLR, neutrophile-lymphocyte ratio.

Supplementary Table 2: Baseline Characteristics according to Ishihara model

|  |  | **n** | **Non-Normalized** | **Normal** | **Normalized** | **p** |
| --- | --- | --- | --- | --- | --- | --- |
| Total Number |  |  | 133 | 108 | 48 | NA |
| Age | Median (Range) |  | 68 (28-91) | 68 (38-86) | 68 (47-84) | 0.778 |
| Gender | Female | 49 | 19 (14.3%) | 20 (18.5%) | 10 (20.8%) | 0.514 |
|  | Male | 240 | 114 (85.7%) | 88 (81.5%) | 38 (79.2%) |  |
| ECOG PS | 0 | 115 | 40 (43%) | 54 (58.7%) | 21 (51.2%) | 0.293 |
|  | 1 | 95 | 44 (47.3%) | 33 (35.9%) | 18 (43.9%) |  |
|  | 2 | 14 | 7 (7.5%) | 5 (5.4%) | 2 (4.9%) |  |
|  | 3 | 2 | 2 (2.2%) | NA | NA |  |
| Child Pugh Score | A | 196 | 78 (62.4%) | 81 (81%) | 37 (82.2%) | 0.01* |
|  | ≥B | 74 | 47 (37.6%) | 19 (19%) | 8 (17.8%) |  |
| Extrahepatic Manifestation | Yes | 96 | 43 (32.3%) | 35 (32.4%) | 18 (37.5%) | 0.800 |
|  | No | 193 | 90 (67.7%) | 73 (67.6%) | 30 (62.5%) |  |
| Prior Local Treatment | Yes | 155 | 63 (47.7%) | 63 (58.3%) | 29 (60.4%) | 0.163 |
|  | No | 133 | 69 (52.3%) | 45 (41.7%) | 19 (39.6%) |  |
| ICI | Atezolizumab/ Bevacizumab | 235 | 113 (85%) | 87 (80.6%) | 35 (72.9%) | 0.035* |
|  | Tremelimumab/Durvalumab | 25 | 9 (6.8%) | 11 (10.2%) | 5 (10.4%) |  |
|  | Nivolumab | 15 | 6 (4.5%) | 2 (1.9%) | 7 (14.6%) |  |
|  | Pembrolizumab | 12 | 5 (3.8%) | 6 (5.6%) | 1 (2.1%) |  |
|  | Other | 2 | NA | 2 (1.9%) | NA |  |
| Baseline AFP | >400 ng/mL | 77 | 45 (37.5%) | 19 (18.6%) | 13 (27.7%) | 0.009** |
|  | ≤400 ng/mL | 192 | 75 (62.5%) | 83 (81.4%) | 34 (72.3%) |  |
| Baseline CRP | >5 mg/L | 181 | 133 (100%) | NA | 48 (100%) | <0.001*** |
|  | ≤5 mg/L | 108 | NA | 108 (100%) | NA |  |
| Baseline NLR | ≥2.7 | 170 | 88 (69.8%) | 55 (57.9%) | 27 (61.4%) | 0.176 |
|  | <2.7 | 95 | 38 (30.2%) | 40 (42.1%) | 17 (38.6%) |  |

Data are Median (Range) or n (%). P-values were calculated using Kruskal-Wallis test for continuous variables and the Pearson chi-square test or Fisher’s exact test for categorical variables.

Abbreviations: AFP, alpha-fetoprotein; CRP, C-reactive protein; ECOG PS, Eastern Cooperative Oncology Group performance status; ICI, immune checkpoint inhibitor; NLR, neutrophile-lymphocyte ratio.

Supplementary Table 3: Detailed overview of univariate and multivariate Cox Regression for PFS

|  |  |  | | **Univariate** | | **Multivariate  Fukuda Model** | | **Multivariate Ishihara Model** | |
| --- | --- | --- | --- | --- | --- | --- | --- | --- | --- |
|  |  | | **n** | **HR (95% CI)** | **p** | **HR (95% CI)** | **p** | **HR (95% CI)** | **p** |
| Age | >60 | | 224 |  |  |  |  |  |  |
|  | ≤60 | | 65 | 0.99 [0.72 - 1.36] | 0.958 |  |  |  |  |
| Gender | Female | | 49 |  |  |  |  |  |  |
|  | Male | | 240 | 0.89 [0.63 - 1.26] | 0.517 |  |  |  |  |
| Child Pugh Score | A | | 196 |  |  |  |  |  |  |
|  | ≥B | | 74 | 1.57 [1.16 - 2.12] | 0.003** | 1.34 [0.87 - 2.06] | 0.18 | 1.41 [0.93 - 2.14] | 0.104 |
| ECOG PS | 0 | | 115 |  |  |  |  |  |  |
|  | 1 | | 95 | 0.88 [0.64 - 1.21] | 0.439 | 0.71 [0.49 - 1.03] | 0.073 | 0.71 [0.49 - 1.02] | 0.062 |
|  | ≥2 | | 16 | 1.89 [1.03 - 3.49] | 0.04* | 1.81 [0.93 - 3.53] | 0.08 | 1.96 [1.01 - 3.81] | 0.048* |
| ICI | Atezo/Bev | | 235 |  |  |  |  |  |  |
|  | Other ICIs | | 54 | 0.88 [0.62 - 1.24] | 0.457 | 0.75 [0.49 - 1.16] | 0.199 | 0.77 [0.5 - 1.18] | 0.231 |
| Baseline CRP | >5 mg/L | | 181 |  |  |  |  |  |  |
|  | ≤5 mg/L | | 108 | 0.68 [0.52 - 0.9] | 0.006** |  |  |  |  |
| Baseline AFP | >400 ng/mL | | 77 |  |  |  |  |  |  |
|  | ≤400 ng/mL | | 192 | 0.63 [0.47 - 0.84] | 0.002** | 0.46 [0.31 - 0.69] | <0.001*** | 0.57 [0.39 - 0.83] | 0.003** |
| Baseline NLR | ≥2.7 | | 170 |  |  |  |  |  |  |
|  | <2.7 | | 95 | 0.91 [0.69 - 1.21] | 0.53 | 1.16 [0.81 - 1.65] | 0.418 | 1.08 [0.76 - 1.53] | 0.668 |
| Fukuda Model | Non-Responder | | 153 |  |  |  |  |  |  |
|  | Responder | | 95 | 0.74 [0.56 - 0.99] | 0.044* | 0.84 [0.56 - 1.25] | 0.393 |  |  |
|  | Flare Responder | | 41 | 0.46 [0.3 - 0.7] | <0.001*** | 0.44 [0.26 - 0.75] | 0.003** |  |  |
| Ishihara Model | Non-Normalized | | 133 |  |  |  |  |  |  |
|  | Normal | | 108 | 0.51 [0.38 - 0.68] | <0.001*** |  |  | 0.45 [0.31 - 0.65] | <0.001*** |
|  | Normalized | | 48 | 0.41 [0.28 - 0.6] | <0.001*** |  |  | 0.37 [0.22 - 0.6] | <0.001*** |

Abbreviations: AFP, alpha-fetoprotein; CRP, C-reactive protein; ECOG PS, Eastern Cooperative Oncology Group performance status; NLR, neutrophile-lymphocyte ratio.

Supplementary Table 4: Detailed overview of univariate and multivariate Cox Regression for OS

|  |  |  | **Univariate** | | **Multivariate  Fukuda Model** | | **Multivariate Ishihara Model** | |
| --- | --- | --- | --- | --- | --- | --- | --- | --- |
|  |  | **n** | **HR (95% CI)** | **p** | **HR (95% CI)** | **p** | **HR (95% CI)** | **p** |
| Age | >60 | 224 |  |  |  |  |  |  |
|  | ≤60 | 65 | 0.98 [0.68 - 1.41] | 0.91 |  |  |  |  |
| Gender | Female | 49 |  |  |  |  |  |  |
|  | Male | 240 | 0.93 [0.63 - 1.38] | 0.714 |  |  |  |  |
| Child Pugh Score | A | 196 |  |  |  |  |  |  |
|  | ≥B | 74 | 2.67 [1.92 - 3.7] | <0.001*** | 2.41 [1.5 - 3.86] | <0.001*** | 2.32 [1.45 – 3.7] | <0.001*** |
| ECOG PS | 0 | 115 |  |  |  |  |  |  |
|  | 1 | 95 | 1.22 [0.83 - 1.77] | 0.31 | 0.96 [0.61 - 1.5] | 0.853 | 0.83 [0.53 - 1.3] | 0.422 |
|  | ≥2 | 16 | 3.21 [1.66 - 6.23] | <0.001*** | 1.66 [0.79 - 3.51] | 0.181 | 1.75 [0.84 - 3.67] | 0.135 |
| ICI | Atezo/Bev | 235 |  |  |  |  |  |  |
|  | Other ICIs | 54 | 0.46 [0.29 - 0.73] | <0.001*** | 0.47 [0.26 - 0.84] | 0.011* | 0.51 [0.29 - 0.91] | 0.023* |
| Baseline CRP | >5 mg/L | 181 |  |  |  |  |  |  |
|  | ≤5 mg/L | 108 | 0.48 [0.34 - 0.68] | <0.001*** |  |  |  |  |
| Baseline AFP | >400 ng/mL | 77 |  |  |  |  |  |  |
|  | ≤400 ng/mL | 192 | 0.63 [0.45 - 0.88] | 0.007** | 0.55 [0.35 - 0.86] | 0.009** | 0.68 [0.43 - 1.07] | 0.094 |
| Baseline NLR | ≥2.7 | 170 |  |  |  |  |  |  |
|  | <2.7 | 95 | 0.67 [0.47 – 0.94] | 0.021* | 0.76 [0.49 - 1.18] | 0.228 | 0.78 [0.5 - 1.2] | 0.252 |
| Fukuda Model | Non-Responder | 153 |  |  |  |  |  |  |
|  | Responder | 95 | 0.66 [0.47 - 0.93] | 0.016* | 0.75 [0.46 - 1.22] | 0.253 |  |  |
|  | Flare Responder | 41 | 0.45 [0.27 - 0.74] | 0.002** | 0.41 [0.21 - 0.82] | 0.011* |  |  |
| Ishihara Model | Non-Normalized | 133 |  |  |  |  |  |  |
|  | Normal | 108 | 0.33 [0.23 - 0.47] | <0.001*** |  |  | 0.32 [0.2 - 0.52] | <0.001*** |
|  | Normalized | 48 | 0.29 [0.18 - 0.46] | <0.001*** |  |  | 0.29 [0.16 - 0.52] | <0.001*** |

Abbreviations: AFP, alpha-fetoprotein; CRP, C-reactive protein; ECOG PS, Eastern Cooperative Oncology Group performance status; NLR, neutrophile-lymphocyte ratio.

Supplementary Table 5: Detailed overview of univariate and multivariate logistic Regression for ORR

|  |  | **Univariate** | | **Multivariate Fukuda Model** | | **Multivariate Ishihara Model** | |
| --- | --- | --- | --- | --- | --- | --- | --- |
|  |  | **OR (95% CI)** | **P Value** | **OR (95% CI)** | **P Value** | **OR (95% CI)** | **P Value** |
| Age | >60 |  |  |  |  |  |  |
|  | ≤60 | 0.63 [0.33 - 1.15] | 0.141 |  |  |  |  |
| Gender | Female |  |  |  |  |  |  |
|  | Male | 0.99 [0.52 - 1.95] | 0.969 |  |  |  |  |
| Child Pugh Score | A |  |  |  |  |  |  |
|  | ≥B | 0.61 [0.33 - 1.08] | 0.099 | 0.85 [0.37 - 1.89] | 0.689 | 1.06 [0.44 - 2.5] | 0.893 |
| ECOG PS | 0 |  |  |  |  |  |  |
|  | 1 | 1.51 [0.86 - 2.67] | 0.151 | 1.34 [0.69 - 2.61] | 0.387 | 1.58 [0.8 - 3.19] | 0.194 |
|  | ≥2 | 0.32 [0.05 - 1.24] | 0.147 | 0.2 [0.01 - 1.15] | 0.137 | 0.19 [0.01 - 1.19] | 0.136 |
| ICI | Atezo/Bev |  |  |  |  |  |  |
|  | Other ICIs | 1.59 [0.86 - 2.91] | 0.131 | 1.26 [0.58 - 2.72] | 0.553 | 1.14 [0.51 - 2.52] | 0.738 |
| Baseline CRP | >5 mg/L |  |  |  |  |  |  |
|  | ≤5 mg/L | 1.95 [1.18 - 3.24] | 0.009** |  |  |  |  |
| Baseline AFP | >400 ng/mL |  |  |  |  |  |  |
|  | ≤400 ng/mL | 1.66 [0.93 - 3.04] | 0.093 | 2.1 [1 - 4.62] | 0.057 | 1.65 [0.75 - 3.73] | 0.22 |
| Baseline NLR | ≥2.7 |  |  |  |  |  |  |
|  | <2.7 | 0.81 [0.47 - 1.38] | 0.445 | 0.77 [0.39 - 1.5] | 0.449 | 0.76 [0.38 - 1.52] | 0.446 |
| Fukuda Model | Non-Responder |  |  |  |  |  |  |
|  | Responder | 1.9 [1.09 - 3.3] | 0.023* | 1.54 [0.74 - 3.19] | 0.249 |  |  |
|  | Flare Responder | 3.44 [1.69 - 7.11] | <0.001*** | 3.3 [1.36 - 8.27] | 0.009** |  |  |
| Ishihara Model | Non-Normalized |  |  |  |  |  |  |
|  | Normal | 3.31 [1.87 - 5.98] | <0.001*** |  |  | 4.63 [2.1 - 10.78] | <0.001*** |
|  | Normalized | 5.11 [2.52 - 10.57] | <0.001*** |  |  | 5.89 [2.33 - 15.66] | <0.001*** |

Abbreviations: AFP, alpha-fetoprotein; CRP, C-reactive protein; ECOG PS, Eastern Cooperative Oncology Group performance status; NLR, neutrophile-lymphocyte ratio.

Supplementary Table 6: Detailed overview of univariate and multivariate Cox Regression for OS and PFS and logistic Regression for ORR according to baseline CRP

|  |  | | **PFS** | | | | **OS** | | | | **ORR** | | | | | |
| --- | --- | --- | --- | --- | --- | --- | --- | --- | --- | --- | --- | --- | --- | --- | --- | --- |
|  |  | | **Univariate** | | **Multivariate** | | **Univariate** | | **Multivariate** | | **Univariate** | | **Multivariate** | | | |
|  |  | | **HR (95% CI)** | **p** | **HR (95% CI)** | **p** | **HR (95% CI)** | **p** | **HR (95% CI)** | **p** | **HR (95% CI)** | **p** | | **HR (95% CI)** | **p** |  |
| Age | | >60 |  |  |  |  |  |  |  |  |  |  | |  |  |  |
|  | | ≤60 | 0.99 [0.72 - 1.36] | 0.958 |  |  | 0.98 [0.68 - 1.41] | 0.91 |  |  | 0.63 [0.33 - 1.15] | 0.141 | |  |  |  |
| Gender | | Female |  |  |  |  |  |  |  |  |  |  | |  |  |  |
|  | | Male | 0.89 [0.63 - 1.26] | 0.517 |  |  | 0.93 [0.63 - 1.38] | 0.714 |  |  | 0.99 [0.52 - 1.95] | 0.969 | |  |  |  |
| Child Pugh Score | | A |  |  |  |  |  |  |  |  |  |  | |  |  |  |
|  | | ≥B | 1.57 [1.16 - 2.12] | 0.003** | 1.53 [1 - 2.32] | 0.048* | 2.67 [1.92 - 3.7] | <0.001*** | 2.54 [1.59 - 4.05] | <0.001*** | 0.61 [0.33 - 1.08] | 0.099 | | 0.88 [0.38 - 1.97] | 0.764 |  |
| ECOG PS | | 0 |  |  |  |  |  |  |  |  |  |  | |  |  |  |
|  | | 1 | 0.88 [0.64 - 1.21] | 0.439 | 0.7 [0.49 - 1.01] | 0.058 | 1.22 [0.83 - 1.77] | 0.31 | 0.87 [0.56 - 1.36] | 0.544 | 1.51 [0.86 - 2.67] | 0.151 | | 1.49 [0.77 - 2.91] | 0.236 |  |
|  | | ≥2 | 1.89 [1.03 - 3.49] | 0.04* | 1.78 [0.92 - 3.46] | 0.088 | 3.21 [1.66 - 6.23] | <0.001*** | 1.74 [0.83 - 3.65] | 0.145 | 0.32 [0.05 - 1.24] | 0.147 | | 0.2 [0.01 - 1.17] | 0.139 |  |
| ICI | | Atezo/Bev |  |  |  |  |  |  |  |  |  |  | |  |  |  |
|  | | Other ICIs | 0.88 [0.62 - 1.24] | 0.457 | 0.75 [0.49 - 1.15] | 0.186 | 0.46 [0.29 - 0.73] | <0.001*** | 0.46 [0.26 - 0.81] | 0.008** | 1.59 [0.86 - 2.91] | 0.131 | | 1.31 [0.61 - 2.8] | 0.482 |  |
| Baseline NLR | | ≥2.7 |  |  |  |  |  |  |  |  |  |  | |  |  |  |
|  | | <2.7 | 0.91 [0.69 - 1.21] | 0.53 | 1.05 [0.74 – 1.48] | 0.792 | 0.67 [0.47 - 0.94] | 0.021* | 0.79 [0.51 - 1.21] | 0.276 | 0.81 [0.47 - 1.38] | 0.445 | | 0.79 [0.4 - 1.52] | 0.481 |  |
| Baseline AFP | | >400 ng/mL |  |  |  |  |  |  |  |  |  |  | |  |  |  |
|  | | ≤400 ng/mL | 0.63 [0.47 - 0.84] | 0.002** | 0.54 [0.37 - 0.79] | 0.001** | 0.63 [0.45 - 0.88] | 0.007** | 0.65 [0.41 – 1.01] | 0.058 | 1.66 [0.93 - 3.04] | 0.093 | | 1.69 [0.8 - 3.7] | 0.177 |  |
| Baseline CRP | | >5 mg/L |  |  |  |  |  |  |  |  |  |  | |  |  |  |
|  | | ≤5 mg/L | 0.68 [0.52 - 0.9] | 0.006** | 0.66 [0.47 - 0.94] | 0.022* | 0.48 [0.34 - 0.68] | <0.001*** | 0.5 [0.32 - 0.79] | 0.003** | 1.95 [1.18 - 3.24] | 0.009** | | 2.25 [1.17 - 4.39] | 0.016* |  |

Abbreviations: AFP, alpha-fetoprotein; CRP, C-reactive protein; ECOG PS, Eastern Cooperative Oncology Group performance status; NLR, neutrophile-lymphocyte ratio.
